# Supplementary material for: Health and ecological risk of heavy metals in agricultural soils related to Tungsten mining in Southern Jiangxi Province, China
Source: PeerJ. 2024 Apr 1;12:e17200. doi: 10.7717/peerj.17200 (PMC10993887; doi:10.7717/peerj.17200)
Supplement: Supplemental Information 1 — Supplement statistical information on heavy metal concentrations. Supplement the correlation coefficient between soil property factors. Supplement the heavy metal Igeo values at different sampling sites. Supplement the migration of heavy metals in rice. Supplement the correlation between soil factors, heavy metal in paddy rice and the translocation factor(TF). Supplement human health risk parameters for heavy metals in rice. [file peerj-12-17200-s001.docx]

Table S1 Heavy metal concentration of soil(mg/kg)

|  | pH | As | Cd | Cu | Cr | Pb | Mo | W | Zn |
| --- | --- | --- | --- | --- | --- | --- | --- | --- | --- |
| Mean values in this study | 5.22 | 33.4 | 0.62 | 40.5 | 64.0 | 57.4 | 10.2 | 95.0 | 104 |
| CV% | 8.43 | 80.2 | 117 | 49.6 | 22.1 | 54.5 | 112 | 96.1 | 49.7 |
| Max | 6.67 | 151 | 3.85 | 110 | 99.20 | 142 | 52.29 | 431 | 289 |
| Min | 3.74 | 3.41 | 0.078 | 17.24 | 21.83 | 14.2 | 0.314 | 4.24 | 37.7 |
| SD | 0.44 | 26.77 | 0.73 | 20.09 | 14.15 | 31.29 | 11.42 | 91.23 | 51.71 |
| National average values in soils | 6.5 | 9.20 | 0.07 | 20.0 | 53.9 | 23.6 | 1.20 | 2.22 | 67.7 |
| The average background values (ABV) of Jiangxi Province | / | 10.4 | 0.10 | 20.8 | 48.0 | 32.1 | 0.30 | 4.93 | 69.0 |
| Chinese soil criteria (Grade II) | / | 30 | 0.3 | 50 | 150 | 250 | / | / | 200 |
| Dutch target values | / | 29 | 0.8 | 36 | 100 | 85 | 3 | / | 140 |
| Chifeng, North of China | 6.97 | 248.6 | 3.33 | 67.13 | 45.58 | 388.6 | 5.61 | / | 473.7 |
| Dayu, South of China | 5.4 | 59.0 | 1.60 | 51.7 | 74.8 | 77.3 | / | 80.3 | 327.5 |
| Lianhua mountain, South of China | / | 72.7 | 0.18 | 40.7 | 36.3 | 90.7 | 1.51 | 22.3 | 125.6 |
| Baikal (residential area), Russia | 5.99 | 148 | 42.0 | 6.18 | 157 | 0.59 | 318 | 55.0 | 2.10 |

Table S2 Correlation coefficients of soil properties

|  | Fe_2_O_3_ | K_2_O | Na_2_O | CaO | CEC | pH | TOC |
| --- | --- | --- | --- | --- | --- | --- | --- |
| Fe_2_O_3_ | 1 |  |  |  |  |  |  |
| K_2_O | **-0.393^**^** | 1 |  |  |  |  |  |
| Na_2_O | **-0.243^*^** | **0.741^**^** | 1 |  |  |  |  |
| CaO | -0.035 | **0.266^*^** | **0.384^**^** | 1 |  |  |  |
| CEC | 0.014 | -0.144 | **-0.246^*^** | **0.432^**^** | 1 |  |  |
| pH | 0.144 | 0.071 | 0.211 | **0.514^**^** | 0.024 | 1 |  |
| TOC | 0.138 | -0.172 | **-0.366**** | 0.077 | **0.507^**^** | -0.143 | 1 |

Note: ** Correlation is signiﬁcant at the 0.01 level(2-tailed). * Correlation is signiﬁcant at the 0.05 level(2-tailed).

Table S3 Geo-accumulation index( *I_geo_*) of heavy metal in soil in sampling sites

| Site | As | Cd | Cr | Cu | | Pb | | Mo | | W | | Zn | |  |
| --- | --- | --- | --- | --- | --- | --- | --- | --- | --- | --- | --- | --- | --- | --- |
|  | *I_geo_* | *I_geo_* | *I_geo_* | | *I_geo_* | | *I_geo_* | | *I_geo_* | | *I_geo_* | | *I_geo_* | |
| FJ-N | 0.94 | 1.06 | 0.06 | | 0.12 | | -0.30 | | 3.02 | | 3.30 | | -0.24 | |
| FJ-S | 1.35 | 2.43 | -0.16 | | 0.68 | | 0.71 | | 4.06 | | 4.07 | | 0.40 | |
| HL | 0.22 | 0.67 | -0.27 | | -0.03 | | -0.27 | | 2.12 | | 2.00 | | -0.49 | |
| QL | 1.16 | 1.82 | -0.25 | | 0.54 | | 0.28 | | 3.45 | | 3.04 | | 0.11 | |
| CJ-W | -0.45 | 0.36 | -0.36 | | -0.31 | | -0.21 | | 1.18 | | 1.19 | | -0.69 | |
| CJ-E | -0.02 | 0.70 | -0.40 | | -0.23 | | -0.54 | | 1.72 | | 1.45 | | -0.63 | |
| Max | 1.35 | 2.43 | 0.06 | | 0.68 | | 0.71 | | 4.06 | | 4.07 | | 0.40 | |
| Min | -0.45 | 0.36 | -0.40 | | -0.31 | | -0.54 | | 1.18 | | 1.19 | | -0.69 | |
| Mean | 0.53 | 1.17 | -0.23 | | 0.13 | | -0.05 | | 2.59 | | 2.51 | | -0.26 | |
| SD | 0.72 | 0.79 | 0.17 | | 0.41 | | 0.46 | | 1.10 | | 1.14 | | 0.44 | |

Table S4 Translocation factor(TF) values of heavy metal in paddy rice

| Site | As | Cd | Cr | Cu | Mo | Pb | Zn |
| --- | --- | --- | --- | --- | --- | --- | --- |
| FJ-N | 8.75E-03 | 0.623 | 1.07E-03 | 0.111 | 6.37E-04 | 1.28E-03 | 0.198 |
| FJ-S | 5.09E-03 | 2.20 | 1.24E-03 | 0.099 | 7.15E-04 | 8.99E-04 | 0.162 |
| HL | 3.84E-03 | 2.48 | 1.41E-03 | 0.127 | 1.34E-03 | 6.56E-04 | 0.166 |
| QL | 1.20E-02 | 5.34 | 1.41E-03 | 0.195 | 3.46E-03 | 1.08E-03 | 0.306 |
| CJ-W | 2.44E-03 | 0.352 | 1.43E-03 | 0.080 | 1.62E-04 | 5.81E-04 | 0.130 |
| CJ-E | 5.31E-03 | 2.58 | 1.65E-03 | 0.133 | 2.73E-03 | 1.62E-03 | 0.226 |

Table S5 The correlation between soil factors, heavy metal in paddy rice and the translocation factor(TF).

|  | As | Cd | Cr | Cu | Mo | Pb | Zn |
| --- | --- | --- | --- | --- | --- | --- | --- |
| TF | | | | | | | |
| pH | 0.208 | -0.154 | -0.216 | 0.164 | 0.032 | 0.098 | 0.132 |
| TOC | -0.082 | -0.209 | -0.145 | -0.376 | -0.096 | -0.075 | -0.313 |
| K_2_O | 0.124 | **0.404^*^** | **0.414^*^** | 0.314 | 0.276 | 0.277 | 0.239 |
| Na_2_O | 0.089 | **0.526^**^** | **0.394^*^** | 0.313 | 0.207 | 0.234 | 0.229 |
| CaO | 0.267 | 0.153 | 0.067 | 0.227 | 0.270 | -0.071 | 0.222 |
| CEC | 0.025 | -0.007 | -0.018 | -0.149 | -0.086 | -0.282 | -0.138 |
| Fe_2_O_3_ | .407^*^ | 0.255 | 0.002 | 0.222 | 0.059 | 0.074 | 0.323 |
| Heavy metal in paddy rice | | | | | | | |
| pH | **0.424^*^** | -0.001 | -0.167 | 0.237 | 0.143 | -0.030 | 0.189 |
| TOC | 0.377 | 0.000 | 0.155 | -0.052 | **0.422^*^** | **0.464^*^** | 0.325 |
| K_2_O | 0.095 | **0.482^*^** | 0.285 | 0.378 | 0.254 | 0.117 | -0.063 |
| Na_2_O | 0.161 | **0.630^**^** | 0.217 | **0.445^*^** | 0.382 | 0.281 | 0.144 |
| CaO | 0.052 | 0.182 | -0.106 | 0.058 | 0.131 | -0.093 | -0.016 |
| CEC | 0.030 | -0.087 | 0.053 | -0.321 | -0.195 | -0.255 | -0.145 |
| Fe_2_O_3_ | 0.131 | 0.190 | -0.230 | 0.252 | -0.165 | 0.023 | 0.335 |

Notes: ** Correlation is signiﬁcant at the 0.01 level(2-tailed). * Correlation is signiﬁcant at the 0.05 level(2-tailed).

Table S6 The human health risk parameters of heavy metals of paddy rice

| Site | As | | Cd | | Cr | | Pb | | Cu | Mo | Zn | HI | TCR |
| --- | --- | --- | --- | --- | --- | --- | --- | --- | --- | --- | --- | --- | --- |
|  | HQ | CR | HQ | CR | HQ | CR | HQ | CR | HQ | HQ | HQ |  |  |
| FJ-N | 4.03 | 7.77E-04 | 2.41 | 6.31E-03 | 0.187 | 1.20E-04 | 0.093 | 1.35E-06 | 0.729 | 6.55E-03 | 0.465 | 7.92 | 7.21E-03 |
| FJ-S | 3.32 | 6.39E-04 | 7.99 | 2.09E-02 | 0.189 | 1.21E-04 | 0.130 | 1.90E-06 | 0.944 | 1.27E-02 | 0.482 | 13.07 | 2.17E-02 |
| HL | 2.14 | 4.12E-04 | 3.61 | 9.43E-03 | 0.188 | 1.21E-04 | 0.067 | 9.84E-07 | 0.774 | 6.28E-03 | 0.351 | 7.13 | 9.97E-03 |
| QL | 2.48 | 4.78E-04 | 5.61 | 1.47E-02 | 0.177 | 1.14E-04 | 0.062 | 8.98E-07 | 0.794 | 5.53E-03 | 0.394 | 9.52 | 1.53E-02 |
| CJ-W | 1.63 | 3.15E-04 | 0.77 | 2.02E-03 | 0.226 | 1.46E-04 | 0.053 | 7.67E-07 | 0.652 | 1.52E-03 | 0.349 | 3.69 | 2.48E-03 |
| CJ-E | 2.47 | 4.77E-04 | 3.18 | 8.32E-03 | 0.245 | 1.57E-04 | 0.097 | 1.41E-06 | 0.705 | 6.87E-03 | 0.410 | 7.12 | 8.96E-03 |
