# Supplementary material for: Health and ecological risk of heavy metals in agricultural soils related to Tungsten mining in Southern Jiangxi Province, China
Source: PeerJ. 2024 Apr 1;12:e17200. doi: 10.7717/peerj.17200 (PMC10993887; doi:10.7717/peerj.17200)
Supplement: Supplemental Information 2 — The little colour blocks are samples of soil with defferent concentrations. The black triangles are sampling sits in study regin. The red five-pointed stars are tungsten mining aeras. The river is represented by blue curve. Other curves represent administrative areas on the map. The towns where the sampling areas are located are identified by different colors, for example, violet represents Fujiang Town, lilac represents Huanglong Town, green represents Qinglong Town, and medium green represents Chijiang Town. [file peerj-12-17200-s002.docx]

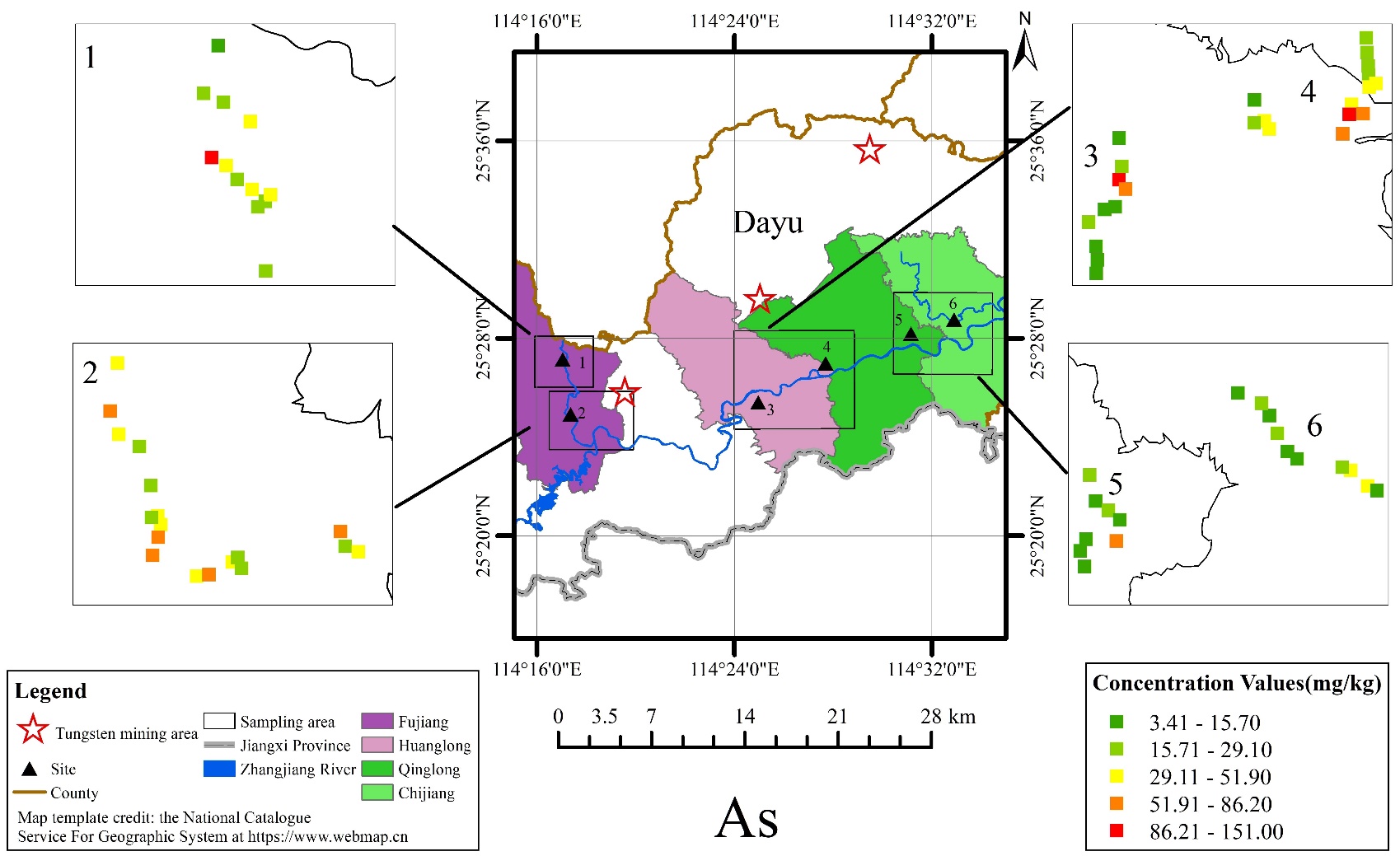


Figure 1S The GIS map on the concentration of Arsenic(As) (site1:FJ-N,site2:FJ-S,site3:HL,site4:QL,site5:CJ-W,site6:CJ-E)


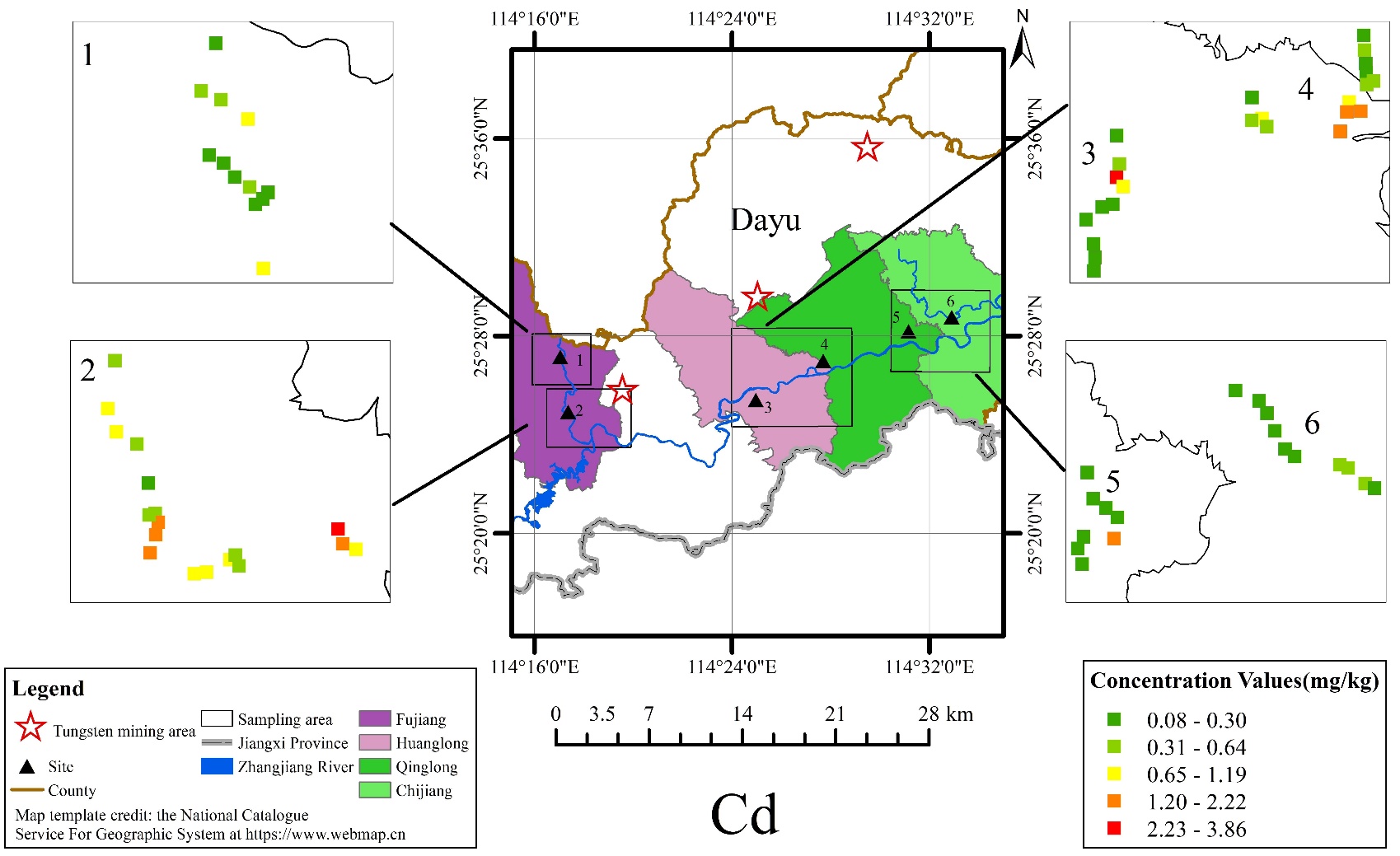


Figure 2S The GIS map on the concentration of Cadmium(Cd) (site1:FJ-N,site2:FJ-S,site3:HL,site4:QL,site5:CJ-W,site6:CJ-E)


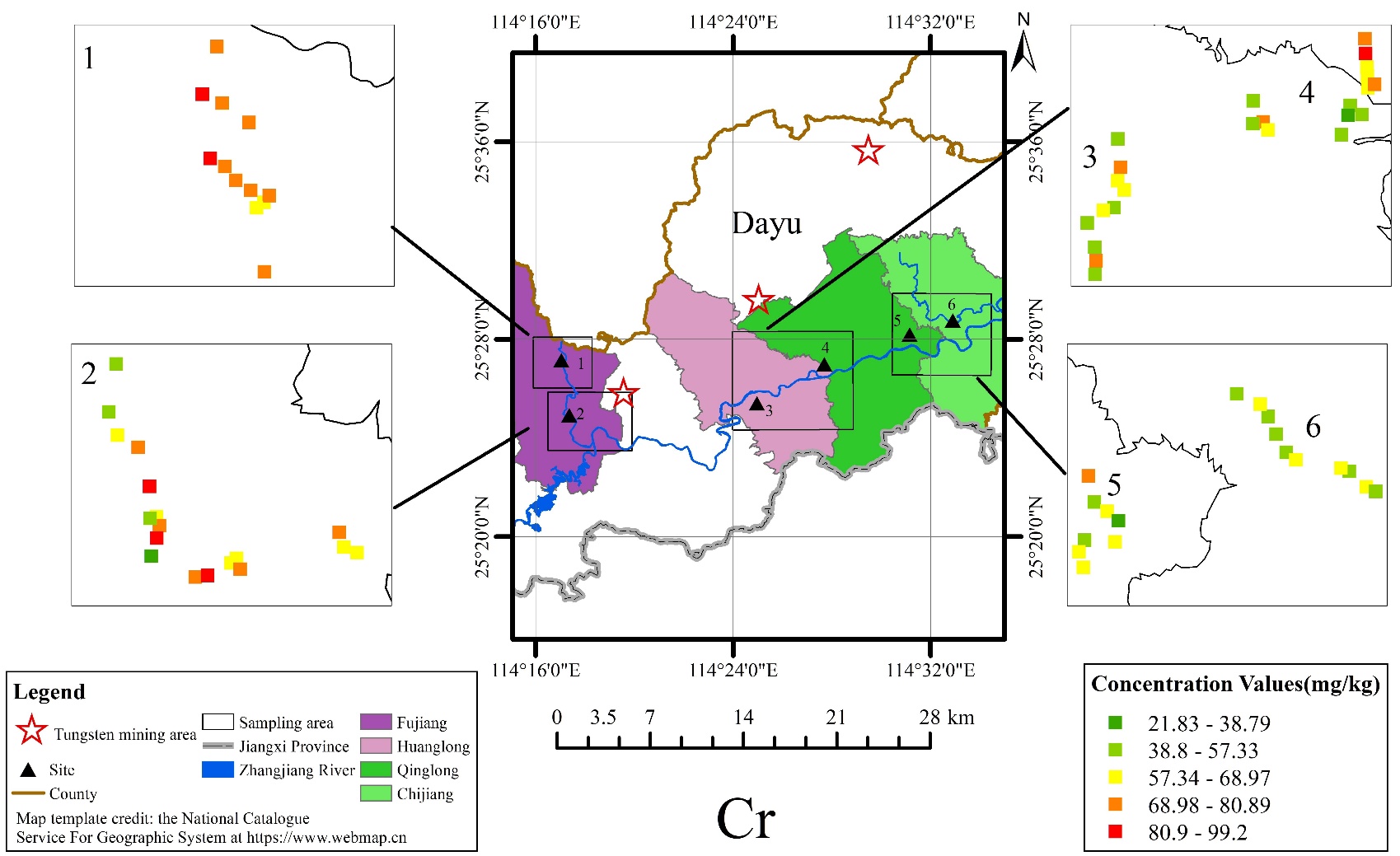


Figure 3S The GIS map on the concentration of Chromium(Cr) (site1:FJ-N,site2:FJ-S,site3:HL,site4:QL,site5:CJ-W,site6:CJ-E)


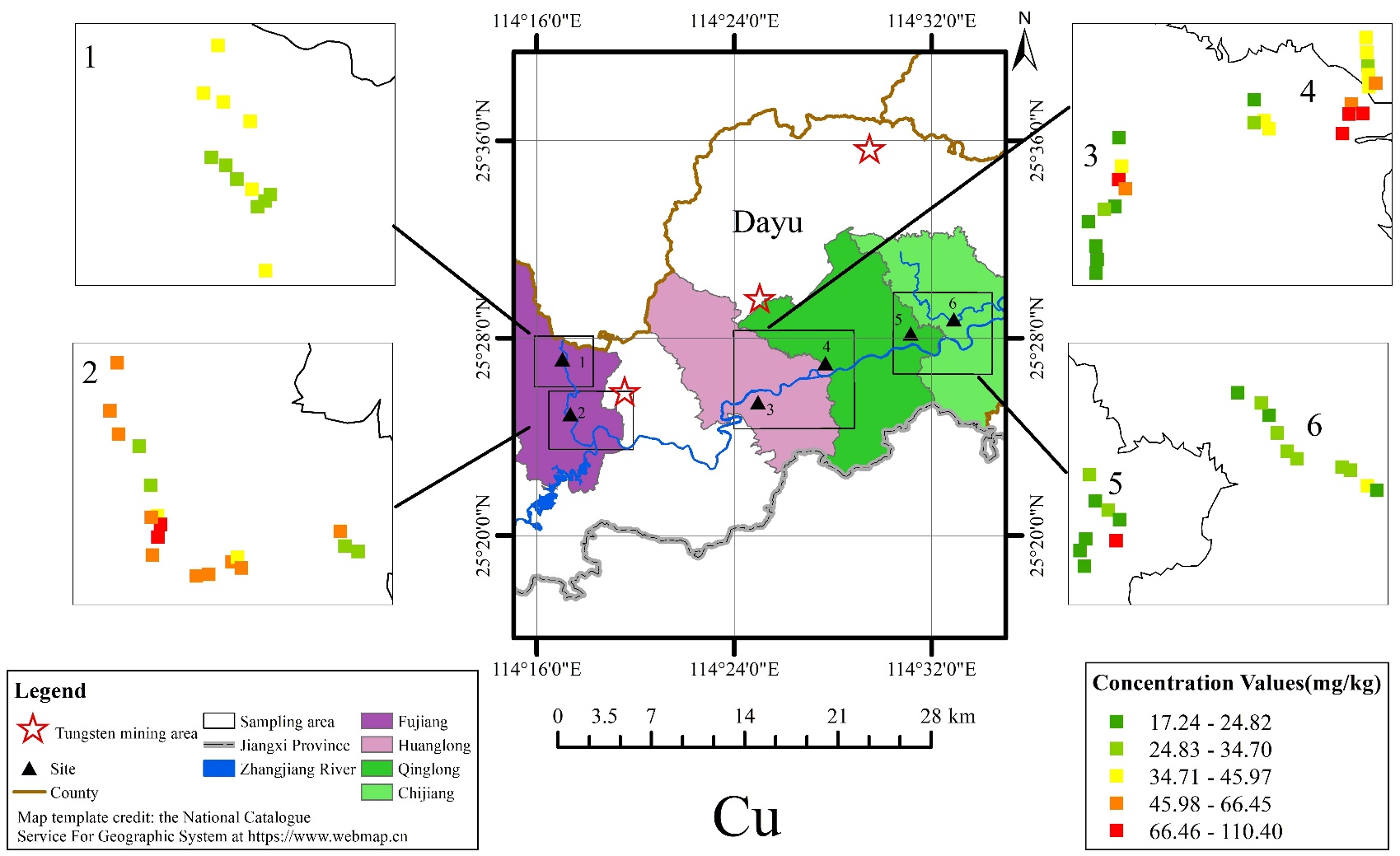


Figure 4S The GIS map on the concentration of Copper(Cu) (site1:FJ-N,site2:FJ-S,site3:HL,site4:QL,site5:CJ-W,site6:CJ-E)


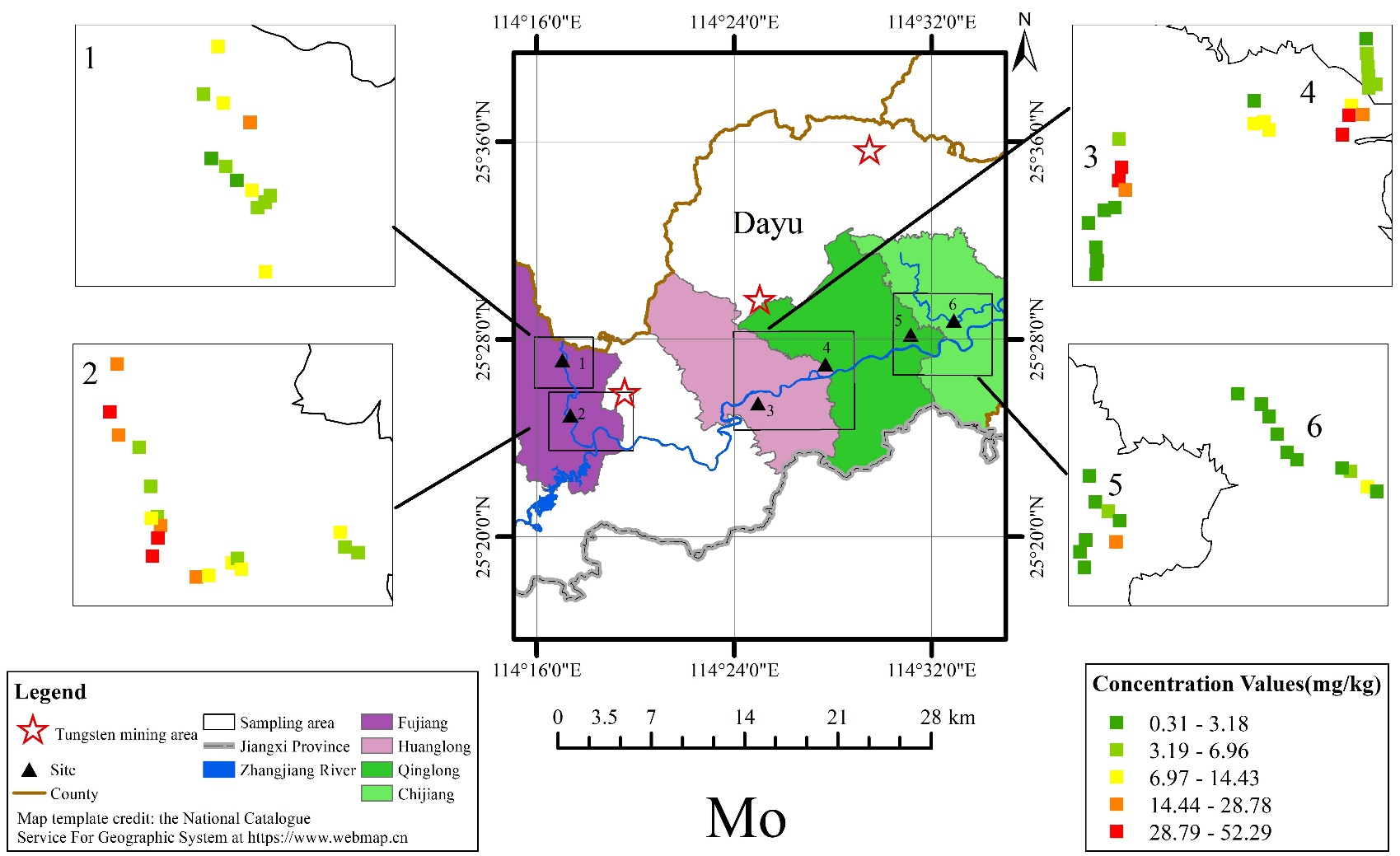


Figure 5S The GIS map on the concentration of Molybdenum(Mo) (site1:FJ-N,site2:FJ-S,site3:HL,site4:QL,site5:CJ-W,site6:CJ-E)


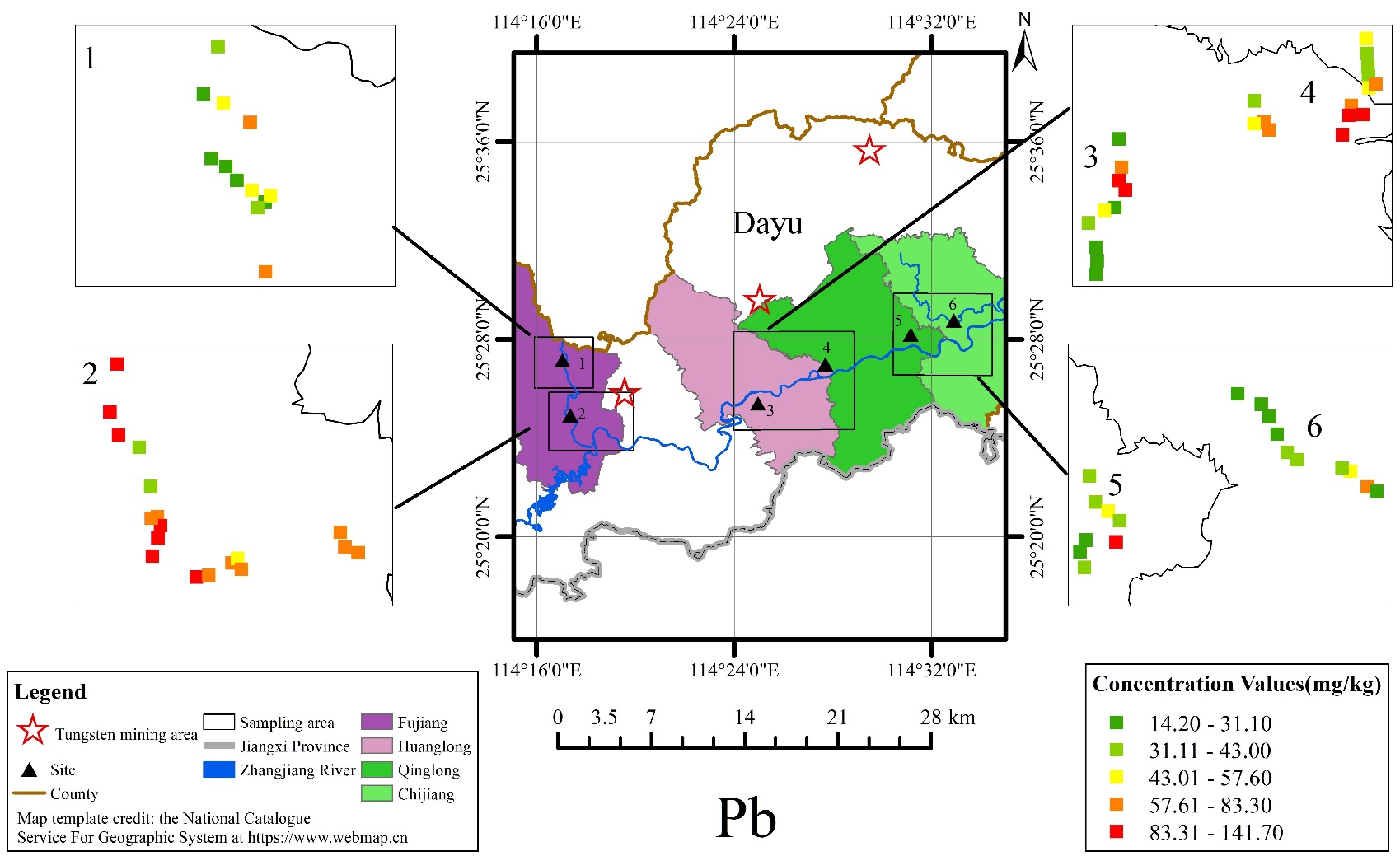


Figure 6S The GIS map on the concentration of Lead(Pb) (site1:FJ-N,site2:FJ-S,site3:HL,site4:QL,site5:CJ-W,site6:CJ-E)


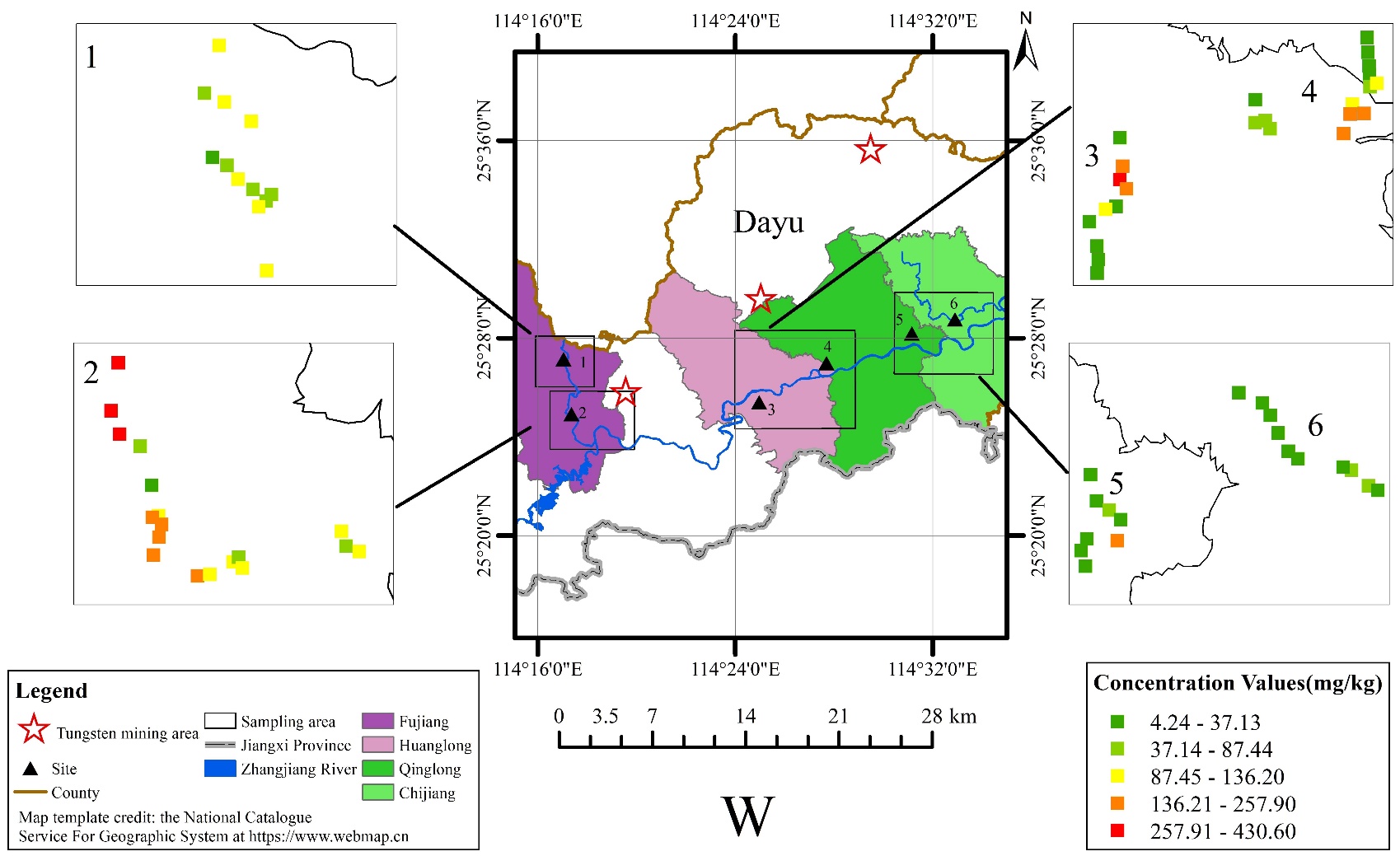


Figure 7S The GIS map on the concentration of Tungsten(W) (site1:FJ-N,site2:FJ-S,site3:HL,site4:QL,site5:CJ-W,site6:CJ-E)


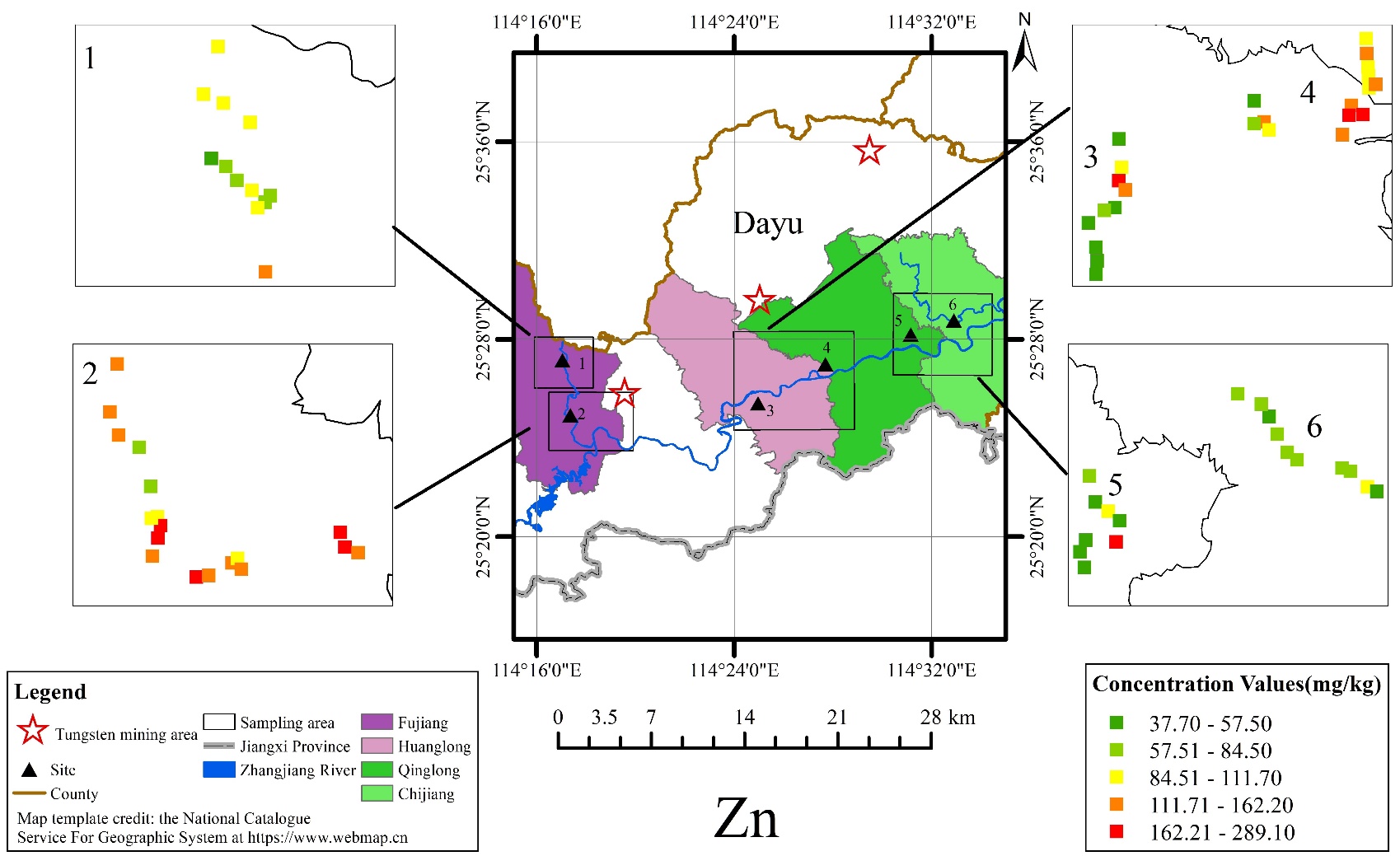


Figure 8S The GIS map on the concentration of Zinc(Zn) (site1:FJ-N,site2:FJ-S,site3:HL,site4:QL,site5:CJ-W,site6:CJ-E)
